# High-Performance and Thermally Robust A_1_-Mode Lamb Wave Resonators on Bonded LiNbO_3_/SiC Membranes

**DOI:** 10.3390/mi16121413

**Published:** 2025-12-15

**Authors:** Noriyuki Watanabe, Shoji Kakio, Yoshiki Sakaida, Hidehiko Oku, Shigeomi Hishiki

**Affiliations:** 1 Integrated Graduate School of Medicine, Engineering, and Agricultural Sciences, University of Yamanashi, Kofu 400-8511, Yamanashi, Japan; kakio@yamanashi.ac.jp; 2 SiC Division, Air Water Inc., 2290-1, Takibe, Toyoshina, Azumino 399-8204, Nagano, Japan; sakaida-yos@awi.co.jp (Y.S.); oku-hid@awi.co.jp (H.O.); hishiki-shi@awi.co.jp (S.H.)

**Keywords:** A_1_-mode Lamb wave resonator, LiNbO_3_/SiC bonded membrane, SiC-on-Si substrate, membrane thermal analysis, thermal stability

## Abstract

In radiofrequency filters, there is an increasing demand for high-frequency, wide-bandwidth operation. Recently, laterally excited A_1_-mode Lamb wave resonators (XBARs) have attracted significant attention; however, freestanding structures are mechanically fragile, limiting their practical implementation. To address this challenge, a novel bonded membrane structure consisting of a lithium niobate (LiNbO_3_; LN) thin plate supported by a silicon carbide (SiC) layer is proposed to realize high-frequency, high-performance, and thermally robust acoustic resonators. Finite element simulations were performed to analyze the excitation and propagation of A_1_-mode Lamb waves in the LN/SiC membrane, clarifying the distinct behavior compared with XBARs. The influence of the bonded SiC thin layer on A_1_-mode Lamb waves was systematically evaluated in terms of coupling coefficient and phase velocity, and design guidelines were established based on these insights. A fabricated LN/SiC resonator with an interdigital electrode pitch of 12 µm exhibited a clear A_1_-mode response near 1.2 GHz, showing an effective electromechanical coupling coefficient of 24% and a phase velocity exceeding 14,000 m/s. These results demonstrate the feasibility of the bonded LN/SiC membrane as a promising platform for high electromechanical coupling, high-speed, and thermally stable acoustic devices.

## 1. Introduction

In response to the growing demands for higher frequencies and broader bandwidths in wireless communication systems such as 5G, 6G, and Wi-Fi, acoustic-wave devices used in radio-frequency (RF) front-end filters are increasingly required to exhibit high electromechanical coupling, high acoustic velocity, high Q-factor, and satisfactory power durability [1,2]. These devices are broadly classified into surface acoustic wave (SAW) and bulk acoustic wave (BAW) devices [3,4,5,6,7,8,9]. In SAW devices, piezoelectric single-crystal substrates, such as LiTaO_3_ (LT) and LiNbO_3_ (LN), are widely used, and operating frequencies can be increased by decreasing the finger pitch (*p*) of the interdigital transducers (IDTs) patterned on the substrate surface. However, excessively fine IDT pitches increase the risk of electromigration and degrade power durability. Consequently, commercially available SAW devices are typically used in the 0.6–2.2 GHz range, with electromechanical coupling coefficients on the order of approximately 8–9%. Furthermore, their phase velocities are generally below 4000 m/s, which fundamentally limits further IDT miniaturization and makes stable operation above 3 GHz challenging. Several studies have developed devices using hybrid substrates with high acoustic velocities or using higher-order modes [10,11,12,13,14,15,16,17] to overcome this limitation. Particularly, numerous approaches have been proposed such as hybrid and multilayer substrates engineered to excite longitudinal leaky SAW (LLSAW) modes with higher phase velocities than that of conventional leaky SAW (LSAW), typically approximately 1.5 times faster, as well as wave-guiding structures and grooved-electrode configurations designed to selectively excite higher-order harmonics, such as the third harmonic and higher. These efforts have demonstrated that LLSAW devices can achieve electromechanical coupling coefficients of 15–24% at approximately 3.5 GHz with phase velocities of approximately 6000 m/s [11,12]. Third-harmonic devices have achieved electromechanical coupling coefficients of 7–8% at approximately 8.8 GHz [16].

BAW devices, including the film bulk acoustic resonator (FBAR) structure, which employs an air cavity, and the solidly mounted resonator (SMR) structure, which uses multilayer acoustic reflectors, are typically used in the 1.7–3 GHz range. Achieving higher frequencies requires further thinning of the piezoelectric film; however, excessively thin films suffer from mechanical strength, power durability, and electromechanical coupling degradation, making stable operation above 3 GHz difficult. Particularly, as reported by Suzuki et al. [18], when the AlN (or metal-doped AlN) film thickness is reduced to the sub-micrometer range for operation above 5 GHz, (i) the resonance frequency is significantly lowered by the mass-loading of the top and bottom electrodes, (ii) the electrode area must be extremely reduced to satisfy 50 Ω impedance matching, and (iii) the electrodes themselves must be very thin, resulting in increased electrical resistance. Combined these factors lead to *Q*-factor, electromechanical coupling, and power-handling capability reductions, presenting significant challenges for further frequency scaling. To overcome these limitations, research has been conducted on utilizing the excitation of higher-order modes enabled by multilayer piezoelectric structures [18,19]. These studies have demonstrated that high-overtone mode SMRs employing polarization-inverted films can achieve electromechanical coupling coefficients of approximately 4% at approximately 5 GHz [18].

Despite these advancements, achieving wide bandwidths remains challenging, as many of these design approaches cannot simultaneously maintain sufficiently high electromechanical coupling coefficients at elevated frequencies. Recently, researchers have investigated the use of plate acoustic waves (PAWs)—a type of bulk acoustic wave that propagates in the lateral direction of a thin piezoelectric plate—to achieve increased frequencies. These devices typically employ IDT electrodes similar to those of SAW devices and utilize a thin piezoelectric crystal substrate. When a thin plate of a piezoelectric material, such as LT or LN, is made to be freestanding, exposing both the top and bottom surfaces to air, various modes such as Lamb waves and shear-horizontal (SH) waves, as well as their higher-order harmonics, can be excited. Among these modes, several types exhibit both high phase velocities and large electromechanical coupling coefficients [20,21,22,23,24,25,26,27,28,29,30,31,32]. In particular, the A_1_ Lamb mode in LN has attracted considerable attention owing to its strong coupling characteristics. An electromechanical coupling coefficient of 15–30% can be achieved near the optimal Euler angle of (0°, 30°, 0°) in LN, although the value significantly depends on the plate thickness [21]. As the plate thickness decreases, the phase velocity *V*_p_ of the A_1_ mode increases significantly, reaching 12,000–25,000 m/s. Because of its high coupling and ultrahigh velocity, the A_1_ mode is a promising candidate for wideband devices operating in the sub-6 GHz frequency range.

Kadota et al. [21] examined the effect of the second Euler angle in the configuration (0º, *θ*º, 0º) in LN and found that an electromechanical coupling coefficient value of approximately 15% can be obtained over a wide range of *θ*, from 0º to 50º. In particular, at approximately *θ* = 30º, the electromechanical coupling coefficient value of the SH_0_ mode approached zero, suggesting that spurious responses owing to the SH_0_ mode could be suppressed effectively. As the ratio of the electrode pitch to the plate thickness (2*p*/*t*) increases to approximately 15–25 and the metallization ratio (*MR*) decreases to ≤0.3, the group velocity of the A_1_ Lamb mode approaches zero (ZGV), leading to the formation of a standing thickness-shear vibration between the IDT-electrode fingers. The resonators using this standing A_1_ mode were termed the “XBARs” (laterally excited bulk acoustic resonators) [26,27], which has attracted significant attention.

Many of the above studies used freestanding piezoelectric thin plates, such as LN, which have drawbacks, including mechanical fragility and poor thermal dissipation in operation, which make their practical application difficult. Solidly mounted resonator (SMR) configurations for BAW devices have been proposed to overcome these limitations [33,34]. However, in SMR structures, the bottom surface of the piezoelectric layer is mechanically constrained, which inevitably leads to degradation of electromechanical coupling and other performance parameters. Void-layer structures [35,36,37,38], in which localized air cavities are introduced beneath the regions with extended vibrational displacements, have been proposed to minimize performance degradation. The air cavities provide free surfaces for the efficient excitation of plate waves, and the piezoelectric film is bonded to the support substrate in the nodes (regions of minimal displacement). Although these configurations improve mechanical robustness and heat dissipation, they involve complex design and fabrication processes and have not yet been implemented in commercial devices.

In response to these challenges, we propose a novel and simple resonator architecture based on a freestanding piezoelectric thin plate integrated with an SiC thin layer on a cost-effective SiC-on-Si substrate, which exhibits high mechanical strength and excellent thermal conductivity. The SiC-on-Si substrate used in this study was developed by AWI Co., Ltd., one of the authors’ affiliations, and was fabricated by epitaxially growing a 3C-SiC thin film on a Si substrate. This structure has the unique advantages of SiC and the cost benefits of an Si-based platform. It has been reported that the thermal conductivity of such 3C-SiC thin films exceeds that of thin-film diamond [39]. In our previous study [40], we demonstrated the potential of SH_0_-mode resonators based on LT/3C-SiC membrane structures. In the preliminary report on the SH_0_-mode prototype, which consisted of a thin LT/SiC multilayer membrane with wavelength *λ* = 6.4 µm, *h*_LT_ = 0.28 (*h*/*λ*), and *h*_SiC_ = 0.14 (*h*/*λ*), a measured resonance frequency *f*_r_ of 679 MHz and the effective electromechanical coupling coefficient *K*_eff_^2^ of 8.9% were obtained (*K*_eff_^2^ is defined as in (2) in the next section). The *K*_eff_^2^ value of the SH_0_-mode device was approximately 1.6 times that of a conventionally fabricated leaky SAW (LSAW) resonator on a bulk LT wafer.

Building on this concept, the present study focused on utilizing the A_1_-mode Lamb wave in rotated Y-cut LN thin plates, which has garnered significant attention for high-frequency operation, primarily because it offers an intrinsically large electromechanical coupling coefficient—substantially exceeding that of commercially available SAW and BAW devices. Although freestanding A_1_-mode Lamb-wave resonators offer superior electromechanical performance, they inherently suffer from mechanical fragility and limited thermal robustness, resulting in poor power durability. To overcome these issues, we adopted a freestanding LN/3C-SiC membrane structure. Owing to the high stiffness and thermal conductivity of the 3C-SiC layer, this composite structure is expected to improve the mechanical stability and heat dissipation compared with a purely freestanding LN membrane. However, because Lamb-wave excitation is most efficient when both surfaces of the piezoelectric thin-plate are free, the introduction of the SiC membrane naturally raised a concern that bonding SiC to LN could degrade the electromechanical coupling compared to an LN-single structure. To verify this concern, we focused on assessing whether SiC membrane addition compromises the essential A_1_-mode characteristics via simulations and resonator fabrication. Although mechanical robustness was not experimentally evaluated in the present study, our primary objective was to verify that the proposed structure preserves the key performance metrics required as A_1_-mode Lamb-wave devices—particularly maintaining an electromechanical coupling coefficient of at least 15%. Additionally, we adopted our co-authors’ SiC-on-Si platform to implement the LN/3C-SiC membrane structure, which enables a low-cost yet high-performance resonator architecture. Accordingly, herein, we discuss their suitability for next-generation RF filter applications.

## 2. Theoretical Analysis

### 2.1. Analysis Models

**Figure 1:**
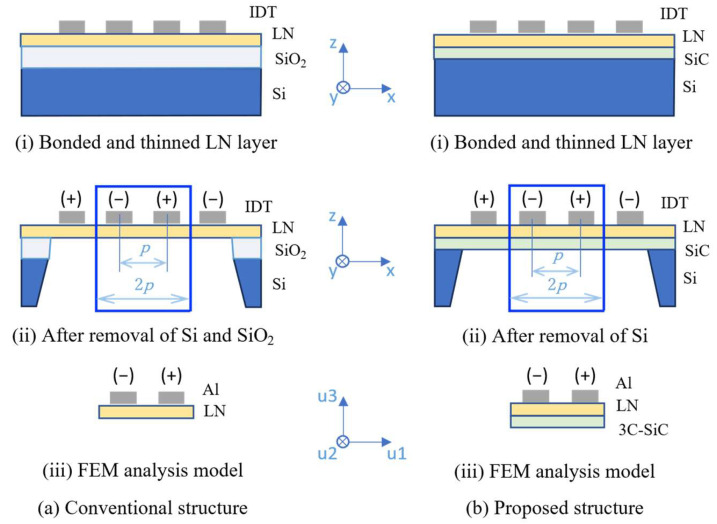
Target structures and finite element method (FEM) models of plate acoustic wave (PAW) resonators with rotated Y-cut LiNbO_3_ (LN).

Two types of freestanding structures were modeled—a conventional LN-single-thin plate and a composite LN/SiC configuration developed in this study—to analyze the electromechanical behavior of the proposed device. The general layouts of the conventional and proposed configurations are illustrated in Figure 1a,b, respectively. For reference, the bonded substrate configurations before backside etching—called, LN/SiO_2_/Si for the conventional structure and LN/SiC/Si for the proposed structure—are depicted in the insets in Figure 1. However, the fabrication processes are not discussed in detail here.

For the finite element method (FEM) analysis, a periodic unit cell with a length of 2*p* was extracted in the u_1_ direction. Here, *p* is the pitch of the IDT fingers, as commonly defined in conventional SAW devices (insets of Figure 1). In the FEM simulations, *p* was initially set to 4.0 µm (that is, 2*p* = 8.0 µm). The thickness of the IDT electrodes was defined as 8% of *p* (that is, 4% of 2*p*). The metallization ratio (*MR*) was set to 0.50, and the thicknesses of both the LN and SiC layers were each set to 0.20*p* (that is, 0.10 of 2*p*).

In Lamb-wave resonators that are driven by IDTs, similar to conventional SAW resonators, structural dimensions are typically normalized using the wavelength, *λ*, defined by the IDT pitch, *λ* = 2*p*. However, in the A_1_ mode, the plate thickness becomes the governing parameter under conditions in which the group velocity approaches zero, corresponding to an effective wavelength of *λ* = 2*t*. To avoid confusion arising from these two definitions of *λ*, we followed the common convention adopted in previous studies by expressing all normalized dimensions in terms of the IDT pitch (2*p*) rather than *λ*.

The piezoelectric material was set to 126ºY-LN, which yielded the maximum effective electromechanical coupling coefficient for the A_1_ mode, as described in Section. The IDT electrodes were made of Al. The SiC layer in the composite LN/SiC thin plate was assumed to be epitaxially 3C-SiC grown on a (111) surface, maintaining its crystallinity. The material quality factors (*Q*ₘ) of all layers were set to 1000, and dielectric losses were ignored in the analysis. Periodic boundary conditions were applied in the u1 direction. A thickness of 50*p* was adopted to incorporate the effect of the aperture length in the depth (u2) direction, and the model was treated as quasi-2.5D.

**Figure 2:**
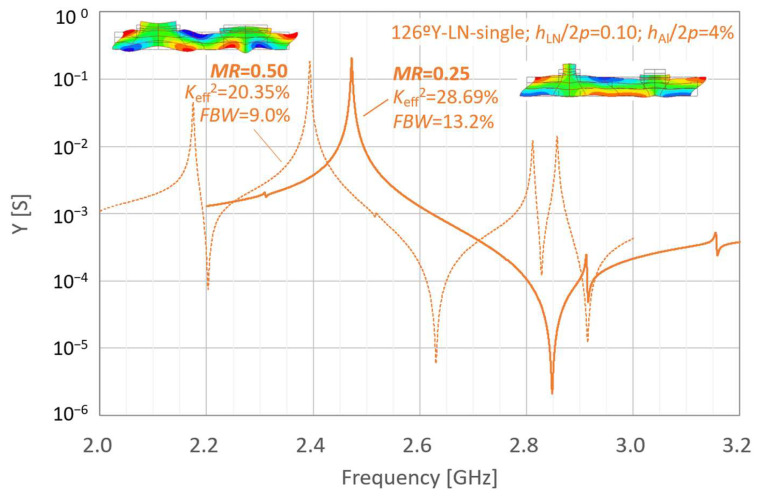
Simulated resonance properties of the LiNbO_3_ (LN) single freestanding resonators under the initial conditions.

**Figure 3:**
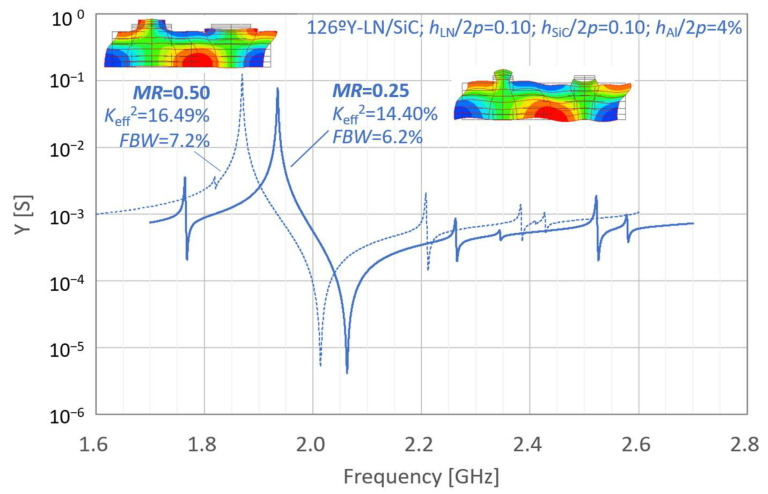
Simulated resonance properties of the LiNbO_3_ (LN)/SiC membrane resonators under the initial conditions.

Figure 2 and Figure 3 present the simulated properties of the LN-single freestanding and LN/SiC membrane resonators at different frequencies under the initial parameter settings, respectively. In Figure 2 and Figure 3, the resonant characteristics are indicated by dashed lines. For comparison, the results obtained under the same conditions but with a reduced metallization ratio *MR* of 0.25 are represented by solid lines.

The corresponding fractional bandwidth (*FBW*) and effective electromechanical coupling coefficient *K*_eff_^2^ are indicated in these figures. *FBW* and *K*_eff_^2^ were calculated using Equations (1) and (2), respectively:

(1) $$FBW=\frac{f_{a}-f_{r}}{f_{a}}$$

(2) $$K_{eff}^{2}=\frac{\pi }{2}\frac{f_{r}}{f_{a}}\tan\left(\frac{\pi }{2}\frac{f_{a}-f_{r}}{f_{a}}\right)$$

where *f*_r_ and *f*_a_ are the resonance and anti-resonance frequencies, respectively.

For the initial layer configuration, the LN-single thin-plate configuration with *MR* = 0.25 exhibits higher *FBW* and *K*_eff_^2^ than that with *MR* = 0.50. In contrast, for the LN/SiC membrane structure with the same initial parameters, a decrease in *MR* did not result in an increase in *FBW*.

**Figure 4:**
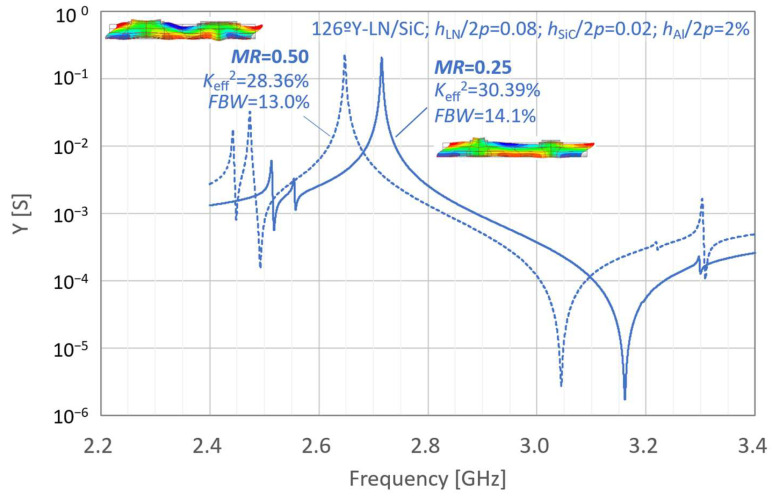
Calculated resonance properties of the LiNbO_3_ (LN)/SiC membrane resonators with the modified configuration.

Figure 4 shows the variations for a modified LN/SiC membrane structure in which the layer thicknesses are adjusted to *h*_LN_/2*p* = 0.08, *h*_SiC_/2*p* = 0.02, *h*_Al_/2*p* = 2%. When the layer thicknesses were adjusted, both *FBW* and *K*_eff_^2^ increased with decreasing *MR*, similar to the trend observed for the LN-single structure. The effects of layer thickness on these characteristics are discussed in detail in the following sections.

### 2.2. Effect of Rotated Y-Cut LN Angle in LN/SiC Membrane Resonator

**Figure 5:**
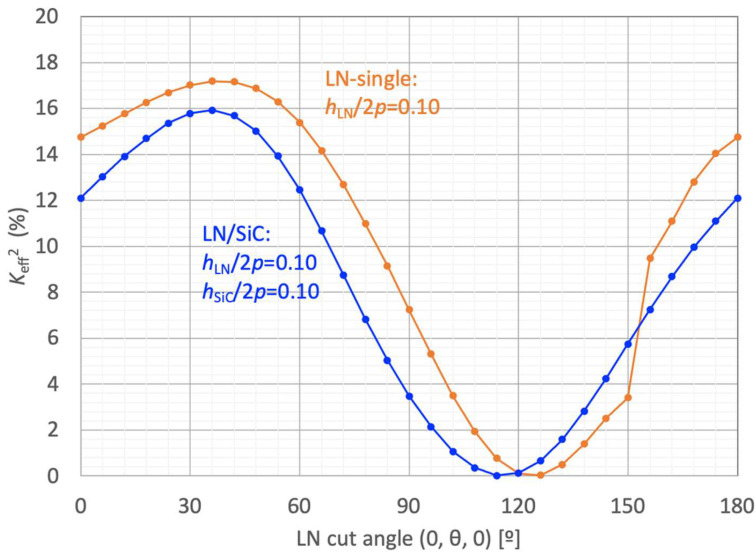
Finite element method (FEM)-calculated effective electromechanical coupling coefficient (*K*_eff_^2^) of the A_1_ mode in the rotated Y-cut LiNbO_3_ (LN) and LN/SiC membranes as a function of Euler angle (0º, *θ*º, 0º).

Figure 5 shows the variations in *K*_eff_^2^ with rotated Y-cut LN angle for the conventional freestanding LN-single structure and the proposed LN/SiC membrane configuration. The results highlight the dependence of A_1_ mode on the second Euler angle (0º, *θ*º, 0º).

The simulation parameters for these two cases are as follows:

(1) LN-single: *h*_LN_/2*p* = 0.10; *h*_Al_/2*p* = 4%; *MR* = 0.50,
(2) LN/SiC: *h*_LN_/2*p* = 0.10; *h*_SiC_/2*p* = 0.10; *h*_Al_/2*p* = 4%; *MR* = 0.50.

For the LN-single structure, a peak *K*_eff_^2^ of 17.2% was obtained at *θ* = 36º–40º (126ºY–130ºY). Under this condition, the resonance frequency *f*_r_ was 2.624 GHz. The corresponding phase velocity *V*_p_(*f*_r_) was obtained using the relation *V*_p_ = 2*p* × *f*_r_ based on the standard convention for SAW and other PAW devices, yielding approximately 21,000 m/s.

For the LN/SiC bonded structure, a peak *K*_eff_^2^ of 15.9% was achieved at *θ* = 36° (126ºY), indicating a slight influence of SiC film bonding compared to that typically observed in SMR-type configurations. The resonance frequency *f*_r_ in this case was 1.860 GHz, and the corresponding phase velocity *V*_p_(*f*_r_) was approximately 14,900 m/s. Although SiC is generally considered a high-velocity material with an acoustic velocity of approximately 10,000 m/s, this value is significantly lower than the phase velocity of the A_1_ mode in the LN-single structure. Therefore, the influence of SiC membrane bonding is more significant on the phase velocity than on the electromechanical coupling coefficient.

### 2.3. Effect of Layer Thickness in the LN/SiC Membrane Resonator

**Figure 6:**
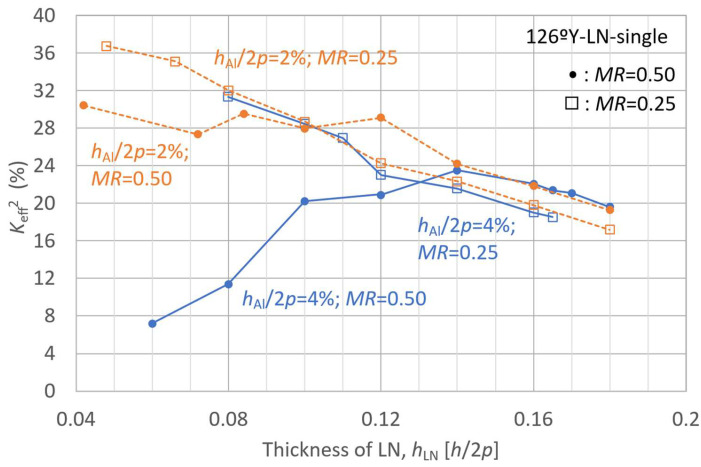
Finite element method (FEM) calculated effective electromechanical coupling coefficient (*K*_eff_^2^) of the A_1_ mode in the LiNbO_3_ (LN)-single membrane as a function of LN layer thickness.

**Figure 7:**
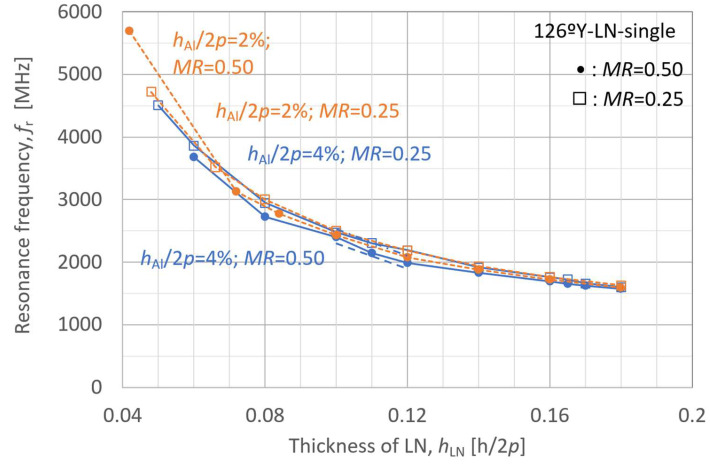
Finite element method (FEM)-calculated resonance frequency (*f*_r_) of the A_1_ mode in the LiNbO_3_ (LN)-single membrane as a function of LN layer thickness.

The behavior of the A_1_-mode *K*_eff_^2^ was first analyzed as a function of the LN plate thickness in the LN-single structure to investigate the influence of individual layer thicknesses. The LN Euler angle *θ* was set to 36° (126°Y), which yielded the maximum *K*_eff_^2^ value presented in Figure 5. The analysis results for *K*_eff_^2^ and *f*_r_ are presented in Figure 6 and Figure 7, respectively, where the LN thickness is normalized with respect to 2*p*.

In Figure 6 and Figure 7, the blue solid lines represent cases in which the IDT electrode thickness is set to an initial value of 4% of 2*p*, whereas the orange dashed lines correspond to a reduced electrode thickness of 2% of 2*p*. The circular markers indicate simulation results for a metallization ratio *MR* of 0.50, and the square markers represent those for *MR* of 0.25.

For the *K*_eff_^2^ behavior of the A_1_ mode in the LN-single plate structure shown in Figure 6, no significant differences due to electrode thickness were observed for *MR* = 0.25, whereas for *MR* = 0.50, the effect of electrode thickness became more pronounced. In contrast, the resonance frequency *f*_r_ shown in Figure 7 exhibited a similar trend for all cases at a macroscopic level.

Furthermore, regarding these behaviors, the square plots for *MR* = 0.25 with *h*_LN_/2*p* below 0.07 are considered to approach a zero group velocity (ZGV) condition, under which the effect of electrode thickness is minimal. Outside the ZGV condition, however, both *K*_eff_^2^ and *f*_r_ are strongly influenced by electrode thickness and *MR*.

**Figure 8:**
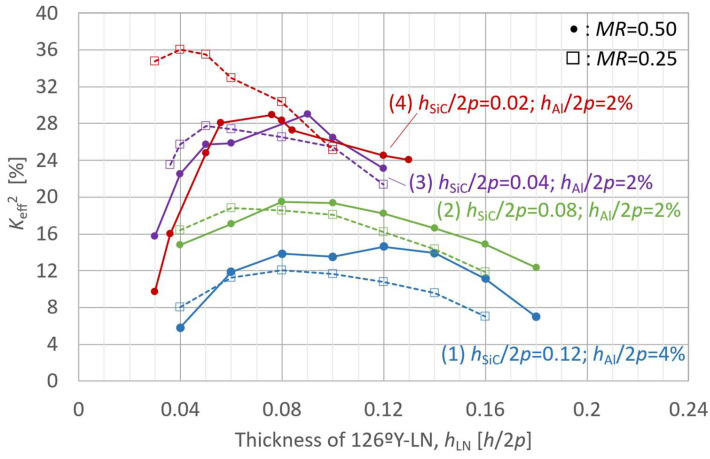
Finite element method (FEM)-calculated effective electromechanical coupling coefficient (*K*_eff_^2^) of the A_1_ mode in LiNbO_3_ (LN)/SiC membrane with various SiC thicknesses, as a function of LN layer thickness.

**Figure 9:**
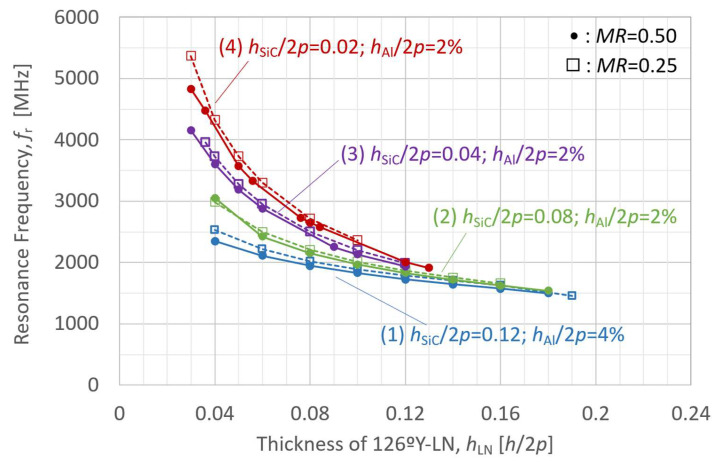
Finite element method (FEM)-calculated resonance frequency (*f*_r_) of the A_1_ mode in LiNbO_3_ (LN)/SiC membrane with various SiC thicknesses, as a function of LN layer thickness.

The simulation results for the effect of the LN layer thickness on the *K*_eff_^2^ and *f*_r_ in the LN/SiC composite structure are presented in Figure 8 and Figure 9, respectively. Figure 8 and Figure 9 present simulation results for four configurations of various layer thicknesses, each evaluated using two different *MR* values (0.50 and 0.25), resulting in a total of eight curves. The pitch was set at 2*p* = 8.0 µm, and the layer parameters other than the LN thickness were set as follows:

(1) 126ºY-LN/SiC: *h*_SiC_/2*p* = 0.12; *h*_Al_/2*p* = 4%;
(2) 126ºY-LN/SiC: *h*_SiC_/2*p* = 0.08; *h*_Al_/2*p* = 2%;
(3) 126ºY-LN/SiC: *h*_SiC_/2*p* = 0.04; *h*_Al_/2*p* = 2%;
(4) 126ºY-LN/SiC: *h*_SiC_/2*p* = 0.02; *h*_Al_/2*p* = 2%.

The circular markers indicate models of *MR* = 0.50, and the square markers correspond to those of *MR* = 0.25.

These behaviors were significantly affected by the thickness of the bonded SiC layer.

The inhibition of the A_1_-mode vibration in *K*_eff_^2^ gradually weakened as the SiC layer became thinner, as shown in Figure 8. For an SiC thickness of *h*_SiC_/2*p* = 0.02 (configuration (4)), the *K*_eff_^2^ behavior with respect to variations in *MR* closely resembled that observed for the LN-single structure shown in Figure 6. However, when the SiC thickness exceeded *h*_SiC_/2*p* = 0.04, the *K*_eff_^2^ peak value for *MR* = 0.50 became comparable to or slightly higher than that for *MR* = 0.25, and the corresponding *h*_LN_ tended to be thicker than that for *MR* = 0.25.

For all SiC thicknesses, the effect of the LN layer thickness on *K*_eff_^2^ showed a gradual and convex profile with a distinct peak. For *MR* = 0.25 (square plots in Figure 8), the peaks of *K*_eff_^2^ were observed at (1) *h*_SiC_/2*p* = 0.12, *h*_LN_/2*p* = 0.08, *K*_eff_^2^ = 12.0%; (2) *h*_SiC_/2*p* = 0.08, *h*_LN_/2*p* = 0.06, *K*_eff_^2^ = 18.8%; (3) *h*_SiC_/2*p* = 0.04, *h*_LN_/2*p* = 0.05, *K*_eff_^2^ = 27.7%; and (4) *h*_SiC_/2*p* = 0.02, *h*_LN_/2*p* = 0.04, *K*_eff_^2^ = 36.0%. As *h*_SiC_/2*p* decreased from 0.12 to 0.02, the peak position shifted toward thinner LN layers, accompanied by an increase in the maximum *K*_eff_^2^ value.

The influence of LN layer thickness on resonance frequency *f*_r_ is predominantly determined by the SiC layer thickness rather than the IDT electrode structure in Figure 9. Furthermore, a thinner SiC layer results in a more pronounced and sensitive increase in resonance frequency *f*_r_ as the LN layer becomes thinner.

**Figure 10:**
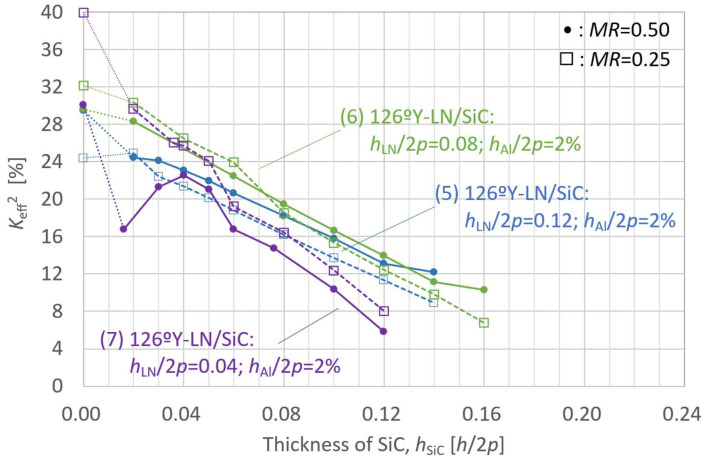
Finite element method (FEM)-calculated effective electromechanical coupling coefficient (*K*_eff_^2^) of the A_1_ mode in the LiNbO_3_ (LN)/SiC membrane with various 126ºY-LN thicknesses, as a function of SiC layer thickness.

**Figure 11:**
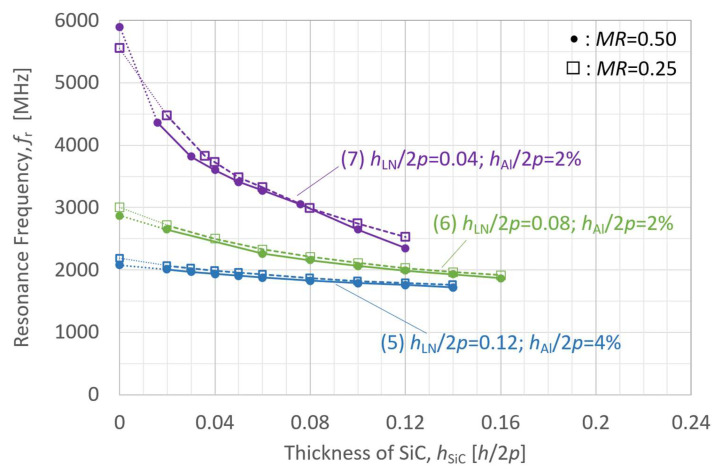
Finite element method (FEM)-calculated resonance frequency (*f*_r_) of the A_1_ mode in the LiNbO_3_ (LN)/SiC membrane with various 126ºY-LN thicknesses, as a function of SiC layer thickness.

Figure 10 and Figure 11 present simulation results for *K*_eff_^2^ and resonance frequency *f*_r_ as functions of the SiC membrane thickness in the LN/SiC structure, respectively. The structural models used in these simulations differ only in SiC thickness, with the other layer parameters set as follows:

(5) 126ºY-LN/SiC: *h*_LN_/2*p* = 0.12; *h*_Al_/2*p* = 2%;
(6) 126ºY-LN/SiC: *h*_LN_/2*p* = 0.08; *h*_Al_/2*p* = 2%;
(7) 126ºY-LN/SiC: *h*_LN_/2*p* = 0.04; *h*_Al_/2*p* = 2%.

For each configuration, simulations were performed for *MR* values of 0.50 and 0.25. 

The variations in *K*_eff_^2^ and *f*_r_ with the SiC layer thickness are shown in Figure 10 and Figure 11, respectively, for three LN layer thickness and two *MR* configurations. All the configurations showed a trend in which *K*_eff_^2^ increased as the SiC layer thickness decreased. However, when the LN layer thickness was *h*_LN_/2*p* = 0.04 (configuration (7)), and *MR* = 0.50, *K*_eff_^2^ began to decrease again when the SiC thickness degreased below *h*_SiC_/2*p* = 0.04. The overall decrease in *K*_eff_^2^ with decreasing LN layer thickness is consistent with the LN thickness dependence observed in Figure 8.

Figure 11 depicts the trend for *f*_r_, where each curve is determined by the LN layer thickness. As the thickness of the LN layer decreased, the variation in *f*_r_ increased, indicating increased sensitivity to the SiC layer thickness.

For clarity, the parameters for the different layer configurations discussed in this section are summarized in Table 1.

### 2.4. Heat Conduction Analysis for the LN/SiC Membrane Structure

Thermal conduction analyses were conducted for thin plate structures of LN-only and LN/SiC to assess the heat dissipation effect of SiC. The analysis model was based on the structures shown in Figure 1a(ii),b(ii), including the Si support substrate. The model assumed 50 pairs of IDT electrodes in the resonator section, with 24 reflector fingers on each side of the resonator, aligned at the same pitch as the IDTs. A half-model with symmetry along the center plane was used. A total heat generation power of 0.5 mW was applied to the IDT electrodes of the resonator, with the bottom surface of the Si substrate fixed at room temperature (25 °C). The resulting temperature increase owing to heat generation was analyzed.

**Figure 12:**
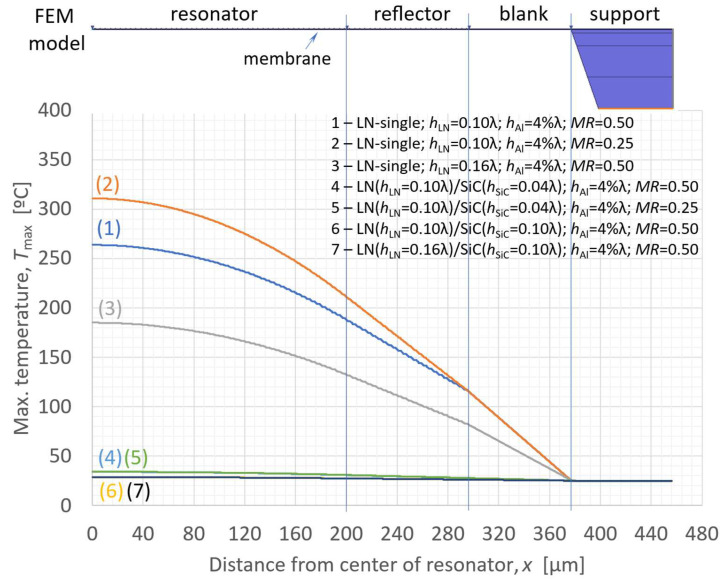
Finite element method (FEM)-calculated heat-generation temperature distribution with various structure configurations.

Figure 12 shows the temperature distribution on the LN surface under the applied heat load. The layer configurations corresponding to each curve in Figure 12 are as follows:

1—LN-single: *h*_LN_/2*p* = 0.10; *h*_SiO2_/2*p* = 0.10; *h*_Al_/2*p* = 4%; *MR* = 0.50
2—LN-single: *h*_LN_/2*p* = 0.10; *h*_SiO2_/2*p* = 0.10; *h*_Al_/2*p* = 4%; *MR* = 0.25
3—LN-single: *h*_LN_/2*p* = 0.16; *h*_SiO2_/2*p* = 0.10; *h*_Al_/2*p* = 4%; *MR* = 0.50
4—LN/SiC: *h*_LN_/2*p* = 0.10; *h*_SiC_/2*p* = 0.04; *h*_Al_/2*p* = 4%; *MR* = 0.50
5—LN/SiC: *h*_LN_/2*p* = 0.10; *h*_SiC_/2*p* = 0.04; *h*_Al_/2*p* = 4%; *MR* = 0.25
6—LN/SiC: *h*_LN_/2*p* = 0.10; *h*_SiC_/2*p* = 0.10; *h*_Al_/2*p* = 4%; *MR* = 0.50
7—LN/SiC: *h*_LN_/2*p* = 0.16; *h*_SiC_/2*p* = 0.10; *h*_Al_/2*p* = 4%; *MR* = 0.50

The thermal conductivities adopted in the simulation were as follows: LN: 3.5 W/(m·K), Al: 237 W/(m·K), Si: 150 W/(m·K), SiO_2_: 1.30 W/(m·K), and 3C-SiC (sheet): 260 W/(m·K) [39].

The failure mechanism of the Al electrodes is attributed to stress migration induced by enhanced grain boundary diffusion. Grain boundary diffusion becomes activated at temperatures of 0.4–0.8 Tm, where Tm is the melting point of the material. Because of the differences in activation energy owing to factors such as crystal orientation, activation tends to commence at the lower end of this range in deposited thin-film electrodes. Therefore, in this study, the critical temperature for activation was assumed to exceed 300 °C, and the input power was selected such that the surface temperature of the LN single-layer structure approached approximately 300 °C.

Although periodic heat generation was applied beneath the IDT electrode fingers, the analysis shown in Figure 12 was conducted under steady-state thermal conduction conditions; thus, no periodic spatial temperature modulation corresponding to the IDT pitch was observed. Instead, the temperature in the thin plate gradually decreased toward the outer edge where the supporting substrate was present. The steady-state analysis represents the saturated thermal accumulation between the electrode fingers, indicating that the maximum heat buildup occurs at the center of the resonator.

According to the analysis results, significant heat accumulation was observed in the LN-single structure, whereas almost no thermal buildup occurred in the LN/SiC thin-membrane structure. These results demonstrate that the SiC layer provides excellent heat dissipation, effectively suppressing thermal accumulation within the resonator and maintaining thermal stability even under high-power operation.

Particularly, while the LN-single structure exhibited a drastic temperature increase, approaching 300 °C under the applied power conditions, the LN/SiC thin-membrane structure exhibited only a modest increase of approximately 30–40 °C. This substantial heat accumulation suppression indicates that thermal-induced failure modes—primarily the melting or rupture of the IDT electrodes, and to a lesser extent potential delamination at the bonded interface—are significantly mitigated in the proposed structure. Consequently, catastrophic failures associated with overheating are expected to be largely eliminated, further underscoring the superior thermal robustness of the LN/SiC resonator.

## 3. Experiments of LN/SiC Membrane Resonators

### 3.1. Prototype of LN/SiC-on-Si Substrate

**Figure 13:**
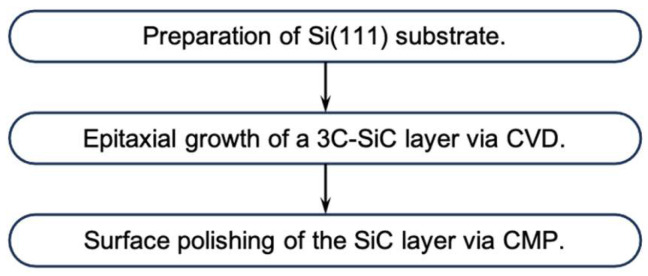
Process flowchart of SiC-on-Si substrate fabrication.

In this study, a SiC-on-Si substrate developed by some of the authors was used to prepare the SiC membrane. The fabrication process of the SiC-on-Si substrate has been described in detail [40]; therefore, only the fabrication flowchart is presented in Figure 13. The fabrication process comprised the following steps: (1) preparation of an Si(111) substrate, (2) epitaxial growth of a 3C-SiC layer on the Si substrate via chemical vapor deposition (CVD), and (3) surface polishing of the grown SiC layer by chemical mechanical polishing (CMP). The fabrication conditions are summarized in Table 2. A 6-in Si (111) wafer was used in this study. Although the standard thickness specified by SEMI and JEITA is 675 or 625 µm, a thicker wafer (thickness: 875 µm) was employed in our prototype fabrication to suppress wafer bowing during the epitaxial growth of the 3C-SiC layer.

Based on the results presented in Figure 5, cut angles of *θ* = 30–42º (corresponding to 120–132ºY in the Y-rotation system) are desirable because the A_1_ mode becomes dominant while the electromechanical coupling of the SH_0_ mode approaches zero in this range. Although 128ºY-rotated LN wafers are commonly used in commercial applications, a 4-in 120ºY-rotated LN wafer was available for this study. Therefore, this wafer was selected and bonded to the SiC-on-Si substrate at room temperature (25 °C).

**Figure 14:**
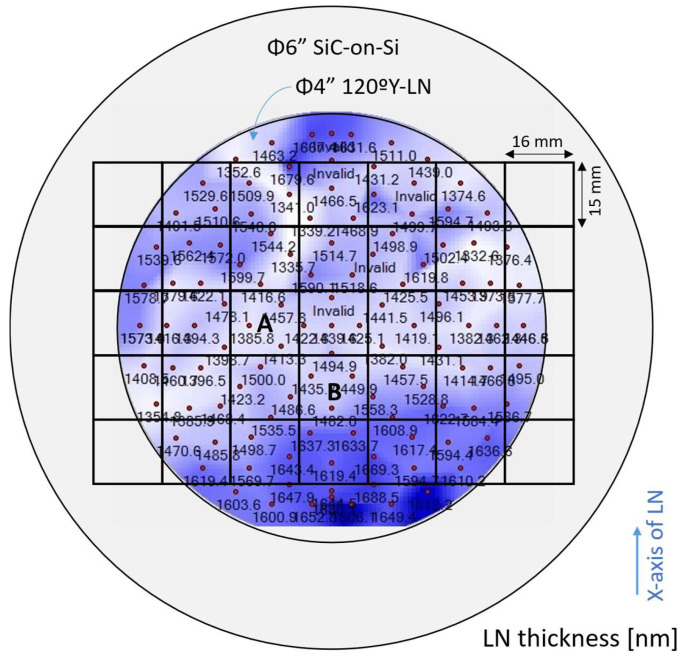
Measured thickness distribution of the thinned LiNbO_3_ (LN) on the SiC-on-Si substrate. Chips A and B correspond to the fabricated samples #A and #B described later, respectively.

Subsequently, the LN layer was thinned down to a thickness of approximately 1.4–1.6 µm. For uniformity characterization after thinning, the LN layer thickness distribution was measured by an optical method, and the results are presented in Figure 14. The thinned LN/SiC/Si wafer was then diced into 16 × 15 mm^2^ pieces. Among these, the regions labeled as chips A and B in Figure 14 were selected and used for samples #A and #B, respectively. The local thickness variation (LTV, defined as 3σ) was 0.09 and 0.12 µm for chips A and B, respectively.

### 3.2. Fabrication of LN/SiC Membrane Resonators

After preparing the LN/SiC-on-Si substrate, the Si-supporting substrate located beneath the resonator region was first coarsely removed via sandblasting, which was used only as a bulk-removal step in a region that does not influence acoustic-wave propagation. As the remaining Si thickness approached the vicinity of the membrane, the process was switched to wet chemical etching to ensure precise and damage-free removal of the final Si layer, ultimately forming the LN/SiC membrane structure.

**Figure 15:**
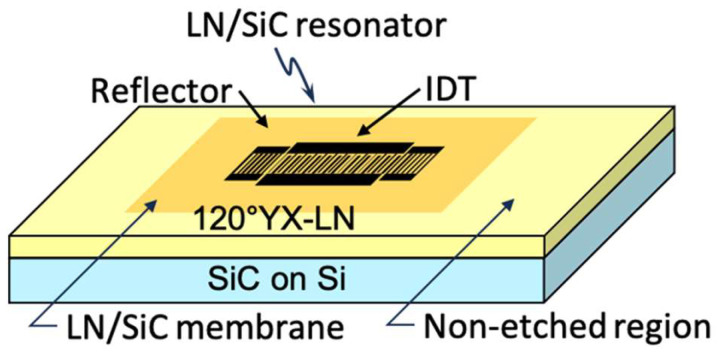
Schematic and optical images of the fabricated LiNbO_3_ (LN)/SiC membrane resonator.

**Figure 16:**
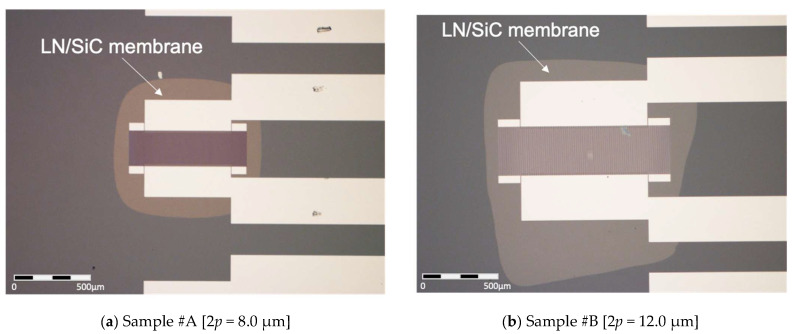
Fabricated LiNbO_3_ (LN)/SiC membrane resonators. #A and #B denote the two fabricated samples (see Table 3 for fabrication conditions).

Subsequently, resonator patterns were formed on the LN surface of the LN/SiC membrane region using photolithography. The patterning conditions are summarized in Table 3. A schematic of the prototype is shown in Figure 15, and a photograph of the fabricated prototype of the LN/SiC membrane resonator is shown in Figure 16. The SiC film thickness used here was a reference value obtained from a separately prepared SiC-on-Si substrate fabricated following the same process.

### 3.3. Measurements for the LN/SiC Membrane Resonators

**Figure 17:**
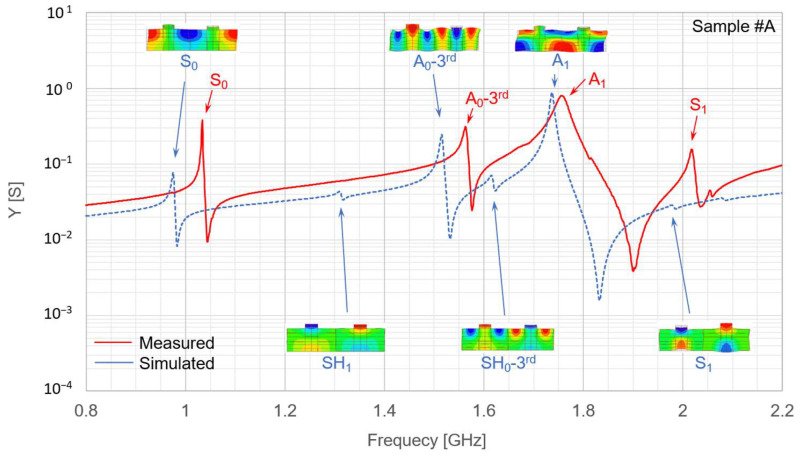
Measured and simulated resonance characteristics of the fabricated LiNbO_3_ (LN)/SiC membrane resonators for Sample #A (interdigital electrode pair pitch 2*p* = 8.0 µm). Sample #A denotes one of the fabricated resonators, as defined in Table 3.

The IDT *MR* is susceptible to variations owing to side-etching during electrode fabrication. Based on the measurements (Figure 16), the *MR* values of Sample #A and #B were approximately 0.28 and 0.36, respectively. The measured responses of the fabricated LN/SiC membrane resonators for Sample #A (2*p* = 8.0 µm) and Sample #B (2*p* = 12.0 µm) are presented in Figure 16 and Figure 17, respectively. The red solid lines represent the measured data, whereas the blue dashed lines indicate the FEM simulation results modeled according to the conditions listed in Table 3.

**Figure 18:**
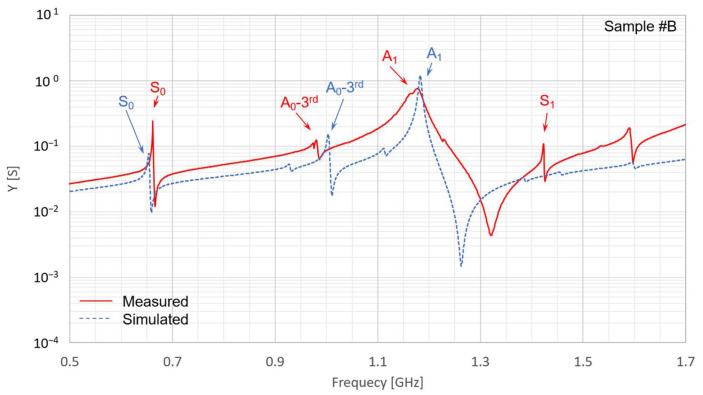
Measured and simulated resonance characteristics of the fabricated LiNbO_3_ (LN)/SiC membrane resonators for Sample #B (interdigital electrode pair pitch 2*p* = 12.0 µm). Sample #B denotes one of the fabricated resonators, as defined in Table 3.

The A_1_ mode observed in the LN/SiC resonators appeared at approximately 1.75 GHz for *K*_eff_^2^ = 17.4% for Sample #A (2*p* = 8.0 µm), and 1.18 GHz for *K*_eff_^2^ = 24.2% for Sample #B (2*p* = 12.0 µm). These effective electromechanical coupling coefficients (17–24%) are approximately two to three times higher than those of commercially available SAW devices, whose typical values are on the order of 8–9%. Each resonance mode in Figure 17 and Figure 18 was identified through comparison with the FEM analysis.

The performance parameters corresponding to the A_1_-mode responses of the LN/SiC membrane resonators are summarized in Table 4. In addition to *K*_eff_^2^, characteristic parameters such as *FBW*, the quality factors at *f*_r_ and *f*_a_ (*Q*_fr_ and *Q*_fa_), and the admittance ratio (*AR*) are also presented in Table 4.

The difference between *f*_r_ and *f*_a_ in the A_1_-mode responses of the fabricated LN/SiC membrane resonators (Samples #A and #B) was higher in the experimental results than in the simulations (Figure 17 and Figure 18). This discrepancy occurred owing to deviations in the SiC and LN layer thicknesses, dimensional variations caused by side etching during IDT fabrication, or slight tapering of the electrode shape. The resulting *K*_eff_^2^ values were approximately 20%, which is approximately twice those of commercially available devices.

However, the measured values of *Q* and *AR* were not satisfactory. As observed for the A_1_-mode resonance peaks in Figure 17 and Figure 18, the resonance profiles lack sharpness. This may be attributed to nonuniformity in the LN layer thickness within the resonator region between the IDT electrodes, which might have caused a dispersion of the resonance frequencies rather than a single concentrated peak. A possible reason for the moderate *Q*-values obtained for the prototypes is the broadened resonance peak. Indeed, based on the LN thickness distribution shown in Figure 14, the LN layer thickness deviation is approximately 3σ = 90 nm. For Sample #A, whose IDT periodicity is 2*p* = 8.0 µm, this corresponds to a normalized thickness difference of Δ*h*_LN_/2*p* ≈ 0.01. When this normalized thickness was varied by 0.01 in the simulation, the A_1_-mode resonance frequency shifted by approximately 45 MHz. Considering that the measured A_1_-mode resonance apex shown in Figure 17 has a width of approximately 20 MHz, the LN thickness nonuniformity can be regarded as one of the contributing factors to the resonance broadening.

Furthermore, this thickness deviation and the 45 MHz frequency shift can also influence the extracted electromechanical coupling coefficient. For sample #A, a deviation corresponding to Δ*h*_LN_/2*p* ≈ 0.01 results in a difference of up to approximately 5% in the simulated coupling coefficient, indicating that the measured-simulated discrepancy in *K*_eff_^2^ may be partly attributed to the LN thickness nonuniformity.

Additionally, returning to the discussion of *Q* degradation, the surface roughening of the SiC layer—which was exposed after etching away the Si support substrate—may also contribute to the *Q* degradation.

Samples #A and #B, which had different IDT pitches, were fabricated from the same wafer; therefore, the layer thicknesses of both samples can be considered nearly identical. The difference observed in their A_1_-mode resonance frequencies thus indicates a strong dependence on the IDT pitch. Similar to other plate-wave modes, the phase velocity *V*_p_ was calculated from the IDT pitch 2*p* using *V*_p_ = *f*_r_ × 2*p*, yielding 14,052 m/s for Sample #A and 14,116 m/s for Sample #B. This result suggests that the resonance frequency of the A_1_ mode can be effectively tuned by adjusting the IDT pitch, which in turn facilitates the frequency separation between series and shunt resonators when designing ladder-type filters. These phase velocities obtained from the IDT periodicity are more than three times higher than those of commercially available SAW devices, whose LSAW velocities are generally approximately 4000 m/s. Additionally, based on the phase velocities obtained in this study, it can be inferred that, assuming an IDT pitch comparable to that used in commercially available SAW devices, a resonance frequency of approximately 9–10 GHz could be achieved, for example, with a pitch of 2*p* = 1.6 µm.

**Figure 19:**
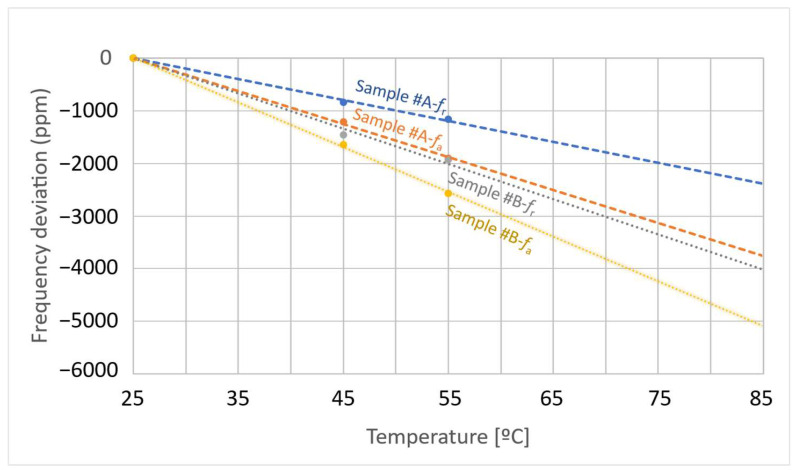
Measured temperature–frequency characteristics of the fabricated LiNbO_3_ (LN)/SiC membrane resonators for Samples #A and #B. Samples #A and #B denote the fabricated sample identifiers (see Table 3).

The temperature characteristics of the fabricated resonators were evaluated at three measurement points, namely, at the reference temperature (25 °C) and two elevated temperatures (45 and 55 °C). At each temperature point, the resonance *f*r and anti-resonance *f*_a_ frequencies were measured, and their deviations from the reference temperature were plotted as shown in Figure 19. The extracted temperature coefficients of frequency (*TCF*) were *TCF*_r_ = −39.7 ppm/°C and *TCF*_a_ = −62.7 ppm/°C for Sample #A, and *TCF*_r_ = −66.9 ppm/°C and *TCF*_a_ = −84.8 ppm/°C for Sample #B. Although Samples #A and #B were fabricated with nearly identical layer thicknesses, the larger IDT pitch of Sample #B results in a smaller normalized structural dimension. Consequently, its resonant characteristics become more sensitive to thermal expansion, leading to larger temperature coefficients absolute values |*TCF*| in Sample #B.

**Figure 20:**
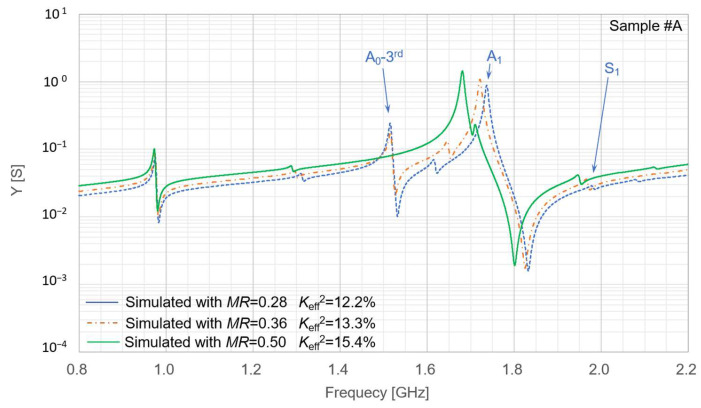
Simulated resonance characteristics obtained by varying the metallization ratio (*MR*) based on the structural parameters of Sample #A. Sample #A denotes one of the fabricated resonators, as defined in Table 3.

In addition to temperature stability, further improvement of spurious-mode suppression is an important design challenge for achieving high-performance resonators. To investigate the behavior of the A_0_-third spurious mode, we performed a simulation analysis based on the layer configuration of Sample #A for different metallization ratio (*MR*) values. The simulated results are indicated in Figure 20. In this example, the A_0_-third spurious mode can be effectively suppressed when *MR* = 0.50. However, as shown in Figure 2, Figure 3 and Figure 4, the comparison between *MR* = 0.50 and 0.25 depends strongly on the specific layer configuration, and additional spurious responses must also be considered. Therefore, *MR* = 0.50 is not always the optimal choice; instead, *MR* should be carefully optimized for each structural condition to achieve the best spurious-mode suppression.

Furthermore, a prominent spurious response owing to the A_0_-third mode appeared immediately before the A_1_-mode resonance in both the fabricated devices and in the simulations. Countermeasures are required to suppress this spurious response, such as fine-tuning the thickness balance of the constituent layers. By contrast, spurious responses attributed to SH modes were effectively suppressed, which is consistent with the prediction based on the selected cut angle.

## 4. Conclusions

As communication systems continue to evolve with each new generation, the demands on RF filter devices are shifting toward higher operating frequencies while simultaneously requiring improved power durability. In this study, an SiC membrane with high hardness and excellent thermal conductivity was developed to improve the performance of acoustic plate-wave devices and compensate for their inherent limitations. The proposed approach for fabricating RF plate-wave devices is cost-effective. Finite element simulations and prototype fabrication were performed to evaluate the excitation and performance of A_1_-mode Lamb waves in the proposed LN/SiC structure. Compared with our previous study on the application of the SH_0_ mode in LT/SiC membranes, we successfully demonstrated the applicability of the A_1_-mode resonator using an LN/SiC membrane structure that offers increased bandwidth and phase velocity. On the basis of detailed FEM analyses and prototype fabrication, the following conclusions are drawn.

(1) FEM analysis revealed that the A_1_ mode in LN/SiC membrane resonators can achieve an extremely high *K*_eff_^2^ value up to approximately 30%, depending on design conditions. Experimental measurements yielded values of approximately 20%.
(2) With the experimental layer configuration, a phase velocity *V*_p_ exceeding 14,000 m/s was obtained. The A_1_ mode in the LN/SiC membrane structure enables frequency tuning through the IDT pitch; for example, resonance frequencies of 1.76 GHz and 1.18 GHz were obtained for pitches of 2*p* = 8.0 µm and 2*p* = 12.0 µm, respectively.
(3) Thermal conduction analysis demonstrated the effectiveness of the high thermal conductivity of SiC in the LN/SiC thin-membrane structure, significantly suppressing temperature rise within the resonator. Notably, even in the very thin-membrane configuration, the peak temperature increase remained as low as only 30–40 °C, in stark contrast to the nearly 300 °C rise observed in the LN-single structure, highlighting its promising potential for high-power durability.
(4) During fabrication and handling of the LN/SiC membrane prototypes, no structural damage was observed, indicating that bonding with the SiC layer contributes to mechanical reinforcement.

The proposed LN/SiC bonded structure is suitable for the development of cost-effective, high-performance, and thermally robust RF acoustic devices. Notably, the demonstrated A_1_-mode resonators exhibit electromechanical coupling coefficients of 17–24%, which are approximately two to three times larger than those of commercially available SAW and BAW devices (7–9%). Furthermore, the extracted phase velocities exceed 14,000 m/s—more than three times higher than typical SAW velocities (<4000 m/s)—highlighting the strong potential of this structure for next-generation high-frequency filter applications.

Future work will focus on suppressing spurious responses near the resonance frequency and further improving the temperature coefficient of frequency, along with evaluating power durability and mechanical robustness, such as shock resistance.

## Figures and Tables

**Table 1:** Simulation conditions for the LiNbO_3_(LN) single and LN/SiC membrane resonators.

Figures		Structure	LN Angle	*h*_LN_/2*p* ^1^	*h*_SiN_/2*p* ^1^	*h*_Al_/2*p* ^1^	*MR*
Figure 2		LN single	126ºY	0.10	–	4.0%	050, 0.25
Figure 3		LN/SiC	126ºY	0.10	0.10	4.0%	050, 0.25
Figure 4		LN/SiC	126ºY	0.08	0.02	2.0%	050, 0.25
Figure 6 and Figure 7		LN single	126ºY	0.04–0.18	–	4.0%	050, 0.25
				0.04–0.18	–	2.0%	050, 0.25
Figure 8 and Figure 9	(1)	LN/SiC	126ºY	0.03–0.18	0.12	4.0%	050, 0.25
	(2)			0.03–0.18	0.08	2.0%	050, 0.25
	(3)			0.03–0.12	0.04	2.0%	050, 0.25
	(4)			0.03–0.12	0.02	2.0%	050, 0.25
Figure 10 and Figure 11	(5)	LN/SiC	126ºY	0.12	0.0–0.14	2.0%	050, 0.25
	(6)			0.08	0.0–0.16	2.0%	050, 0.25
	(7)			0.04	0.0–0.12	2.0%	050, 0.25

^1^ The IDT periodicity is 2*p* = 8.0 µm.

**Table 2:** Fabrication conditions for the SiC-on-Si substrate.

Materials	Items/Process	Conditions
Si substrate	Growth method	MCZ
	Plane	(111)
	Resistivity	>5000 Ωcm
	Wafer size	Φ6”, t875 µm
SiC Deposit	Pre-process	Carbonization of Si substrate surface
	Deposition	3C-SiC epitaxial growth by CVD
	Pressure	Low vacuum (1 × 10^−2^ Pa)
	Temperature	Around 1000 °C
	Material gas	Organosilane gas
	CMP	Ra < 0.7 nm

**Table 3:** Conditions for fabricating LiNbO_3_/SiC membrane resonators. “#A” and “#B” indicate the sample identification numbers of the two fabricated resonators.

		Sample #A [2*p* = 8 µm]	Sample #B [2*p* = 12 µm]
LiNbO_3_ (LN)	Cut-angle	120ºYX (30ºX)	
	Thickness	1.43 µm (0.179 ^1^)	1.48 µm (0.123 ^1^)
SiC	Thickness	0.9 µm (0.1125 ^1^)	0.9 µm (0.075 ^1^)
Electrode	Film material	Al	
	Underlying film	Ti	
	Al thickness	400 nm (5% ^1^)	400 nm (3.3% ^1^)
Resonator	Length of 2*p*	8.0 µm	12.0 µm
	IDT finger	70.5 pairs	
	Aperture width	25 of 2*p*	
	Metallization ratio	0.28	0.36
	Number of reflectors	25 (both sides)	

^1^ The values in parentheses are normalized by 2*p*.

**Table 4:** Comparison of simulated and Measured resonance properties of the fabricated LN/SiC membrane resonators. Samples #A and #B denote the fabricated sample identifiers (see Table 3).

Items	Sample #A [2*p* = 8.0 µm]		Sample #B [2*p* = 12.0 µm]	
	Measured	Simulated	Measured	Simulated
*f*_r_ [GHz]	1.7565	1.7370	1.1764	1.1828
*f*_a_ [GHz]	1.9007	1.8324	1.3207	1.2626
*FBW* (%)	7.59	5.21	10.92	6.32
*K*_eff_^2^ (%)	17.39	12.20	24.24	14.56
*Q*_fr_	68.3	222.7	39.7	219.0
*Q*_fa_	174.1	241.1	89.1	242.8
*AR* (dB)	46.3	54.8	44.9	58.3

## Data Availability

Data available on request due to restrictions.
